# IRC-082451, a Novel Multitargeting Molecule, Reduces L-DOPA-Induced Dyskinesias in MPTP Parkinsonian Primates

**DOI:** 10.1371/journal.pone.0052680

**Published:** 2013-01-03

**Authors:** Romina Aron Badin, Brigitte Spinnewyn, Marie-Claude Gaillard, Caroline Jan, Carole Malgorn, Nadja Van Camp, Frédéric Dollé, Martine Guillermier, Sabrina Boulet, Anne Bertrand, Marc Savasta, Michel Auguet, Emmanuel Brouillet, Pierre-Etienne Chabrier, Philippe Hantraye

**Affiliations:** 1 Molecular Imaging Research Center, Commissariat à l’énergie atomique, Fontenay-aux-Roses, France; 2 URA CEA CNRS 2210, Commissariat à l’énergie atomique, Fontenay-aux-Roses, France; 3 IPSEN Innovation, Les Ulis, France; 4 Service Hospitalier Frédéric Joliot, Institut d’Imagerie BioMédicale – Commissariat à l’énergie atomique, Orsay, France; 5 INSERM U836, Grenoble Institut des Neurosciences Team #10, Grenoble, France; 6 Université Joseph Fourier, Grenoble, France; 7 Centre Hospitalier Universitaire, Grenoble, France; INSERM/CNRS, France

## Abstract

The development of dyskinesias following chronic L-DOPA replacement therapy remains a major problem in the long-term treatment of Parkinson’s disease. This study aimed at evaluating the effect of IRC-082451 (base of BN82451), a novel multitargeting hybrid molecule, on L-DOPA-induced dyskinesias (LIDs) and hypolocomotor activity in a non-human primate model of PD. IRC-082451 displays multiple properties: it inhibits neuronal excitotoxicity (sodium channel blocker), oxidative stress (antioxidant) and neuroinflammation (cyclooxygenase inhibitor) and is endowed with mitochondrial protective properties. Animals received daily MPTP injections until stably parkinsonian. A daily treatment with increasing doses of L-DOPA was administered to parkinsonian primates until the appearance of dyskinesias. Then, different treatment regimens and doses of IRC-082451 were tested and compared to the benchmark molecule amantadine. Primates were regularly filmed and videos were analyzed with specialized software. A novel approach combining the analysis of dyskinesias and locomotor activity was used to determine efficacy. This analysis yielded the quantification of the total distance travelled and the incidence of dyskinesias in 7 different body parts. A dose-dependent efficacy of IRC-082451 against dyskinesias was observed. The 5 mg/kg dose was best at attenuating the severity of fully established LIDs. Its effect was significantly different from that of amantadine since it increased spontaneous locomotor activity while reducing LIDs. This dose was effective both acutely and in a 5-day sub-chronic treatment. Moreover, positron emission tomography scans using radiolabelled dopamine demonstrated that there was no direct interference between treatment with IRC-082451 and dopamine metabolism in the brain. Finally, post-mortem analysis indicated that this reduction in dyskinesias was associated with changes in cFOS, FosB and ARC mRNA expression levels in the putamen. The data demonstrates the antidyskinetic efficacy of IRC-082451 in a primate model of PD with motor complications and opens the way to the clinical application of this treatment for the management of LIDs.

## Introduction

Parkinson’s disease (PD) is the most common neurodegenerative disorder after Alzheimer’s disease affecting the elderly population. The incidence of this neurological disorder dramatically increases with age, with more than 1% of the population over 60 being affected worldwide [1]. Loss of dopaminergic neurons in the substantia nigra pars compacta (SNc) and consequent dopaminergic depletion in the striatum has long been identified as the underlying cause of PD. The result of this loss is reflected by both cognitive and motor decline, with cardinal symptoms being bradykinesia, akinesia, muscular rigidity, postural instability and tremor. The best symptomatological treatment available for this progressive neurodegenerative disorder is replacement therapy with L-DOPA (L-3,4-dihydroxyphenylalanine) [2], [3]. Although this treatment is very efficacious, chronic exposure to the drug rapidly gives rise to fluctuations in motor response and abnormal involuntary movements termed dyskinesias that complicate the management of the disease [4], [5].

The two most widely used animal models of PD that display levodopa-induced dyskinesias (LIDs) under chronic L-DOPA treatment are the intracerebral injection of 6-OHDA in rats and the systemic administration of the neurotoxin MPTP (1-methyl-4-phenyl-1,2,3,6-tetrahydropyridine) in primates respectively. The MPTP monkey model reproduces most of the features seen in PD patients at the behavioural but also biochemical and histological levels [6], [7], [8], [9], [10], [11], [12], [13]. This model has allowed the investigation of the neurophysiological and molecular mechanisms leading to LIDs and the evaluation of potential therapies that could hamper their development or reduce their severity [14], [15]. In this context, we focused our interest on a novel molecule, IRC-082451 (base of BN82451), that belongs to a family of hybrid multitargeting compounds capable of inhibiting lipid peroxydation and of protecting mitochondria against different toxins, reducing mitochondrial swelling, cytochrome c release and caspase-9 activation [16]. In fact, IRC-082451 exhibits anti-inflammatory and anti-oxidant properties both *in vitro* and *in vivo* and displays neuroprotective effects in animal models of Huntington’s disease, cerebral ischemia, PD and amyotrophic lateral sclerosis [16]. The antidyskinetic effect of IRC-082451 has recently been demonstrated in 6-OHDA-lesioned dyskinetic rats [17]. The fact that the mechanism of action of this molecule does not directly involve the dopaminergic or the serotoninergic pathways [16] makes it an interesting alternative to existing PD therapies.

In the present pre-clinical evaluation study, we aimed to evaluate the antidyskinetic properties of IRC-082451 in a primate model of PD with motor complications. The model was first validated with amantadine, an NMDA receptor antagonist with proved efficacy against LIDs [18], [19], [20], [21]. Then, the pharmacological evaluation of IRC-082451 efficacy against LIDs consisted in injection of all dyskinetic animals with three different doses (2.5 to 10 mg/kg) and two administration regimes (acute and sub-chronic), intercalating IRC and vehicle treatments over time. Video recordings were used to perform a comprehensive behavioural characterization of the effects of each dose of IRC-082451 on dyskinetic animals placed under daily L-DOPA maintenance regime and comparing it to the profile of each same animal under vehicle treatment. This approach allowed the precise time-encoded assessment of LIDs and image analysis-based determination of locomotion after L-DOPA administration. L-DOPA concentration measurements and positron emission tomography (PET) scans using 6-[18F] Fluoro-L-Dopa (^18^F-DOPA) were performed to exclude any potential direct interaction of IRC-082451 with the biodistribution/biodisponibility of L-DOPA. Finally, transcriptomic and histological *post mortem* analyses were performed in the putamen of controls, naïve MPTP animals, and MPTP dyskinetic animals under L-DOPA treatment with either IRC-082451 or its vehicle PEG400. Results indicate that IRC-082451 can significantly reduce LIDs in parkinsonian dyskinetic monkeys without compromising the beneficial effect of L-DOPA on locomotion, and reverse some of the striatal molecular anomalies linked to LID.

## Materials and Methods

### Animals and Housing

All animal studies were conducted according European (EU Directive 86/609) and French regulations (French Act Rural Code R 214-87 to 131). The animal facility is authorized by local veterinarian authorities (authorization n° A 92-032-02) and complies with Standards for Humane Care and Use of Laboratory Animals of the Office of Laboratory Animal Welfare (OLAW – n°#A5826-01). All efforts were made to minimize animal suffering and animal care was supervised by veterinarians and animal technicians skilled in the healthcare and housing of non-human primates. All animals were individually housed under standard environmental conditions (12-hour light-dark cycle, temperature: 22±1°C and humidity: 50%) with free access to food and water. Experiments were conducted on a total of twelve male cynomolgus monkeys (*Macaca fascicularis*, supplied by Noveprim, Mauritius Island) of a mean age of 5±1 years and a mean weight of 6±2 kg.

### Experimental Design and Behavioural Analysis

**Figure 1:**
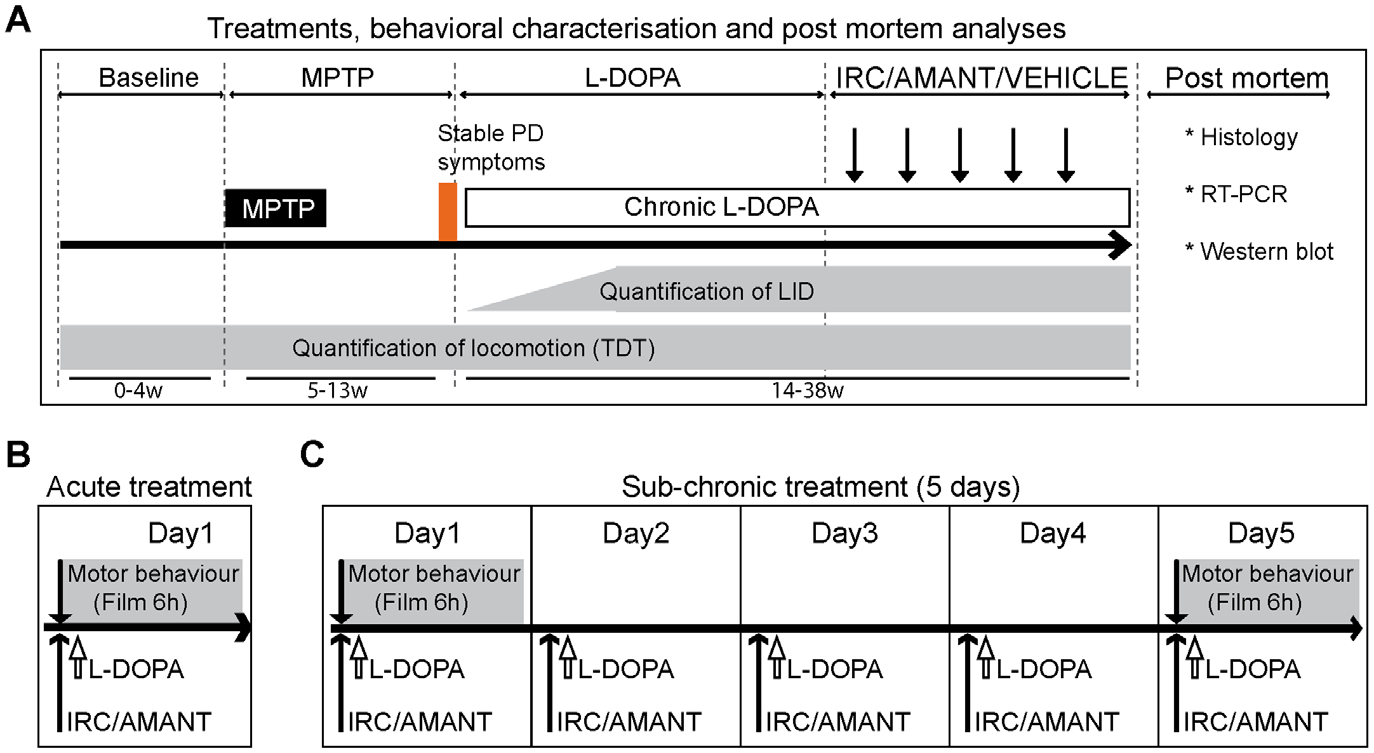
Schematic representation of the experimental design and pharmacological treatment protocols. A, general experimental plan showing phases of behavioural characterization: at baseline, during MPTP intoxication, upon L-DOPA chronic treatment for the development of dyskinesias, and after antidyskinetic treatment administration. Post-mortem analysis techniques are listed. Detail of the administration of L-DOPA and treatment with either IRC-082451 (IRC), amantadine (AMANT) or vehicle (VEH) in acute (B) and sub-chronic (C) treatment regimes.

The general workflow of experiments and the different pharmacological treatment regimens administered are presented in figure 1. Animals were divided into 3 groups: healthy controls (CTRL) (n = 3); MPTP-treated parkinsonian controls (MPTP) (n = 3), MPTP and L-DOPA treated dyskinetic animals (LID) (n = 6). All LID animals were sequentially treated with vehicle PEG400 (VEH), the antidyskinetic molecule IRC-082451 (IRC) or the antidyskinetic molecule of reference amantadine. At euthanasia however, LID animals were sacrificed under L-DOPA and either a 5 day-treatment with IRC (5 mg/kg; n = 3) or vehicle (n = 3). No animal was sacrificed under the effect of amantadine treatment.

IRC-082451 from Ipsen Innovation was obtained by removing HCl from BN82451 and amantadine was purchased from Sigma Aldrich AB.

All animals were videotaped 5 times before the beginning of the protocol in order to quantify and characterize normal baseline locomotor activity with Ethovision® and The Observer® software respectively (Noldus, The Netherlands) (Fig. 1A). Briefly, animals were transported from their home cage to a custom-made video cage without bars and continuously filmed for 6 hours. Baseline locomotor activity was quantified as total distance travelled (TDT) in meters and averaged across all different baseline films using Ethovision [22], [23]. Nine primates received daily intramuscular injections of 0.2 mg/kg MPTP (1-methyl-4-phenyl-1,2,3,6-tetrahydropyridine, Sigma-Aldrich, France) for 7 consecutive days (Fig. 1A), the remaining 3 being kept as intact controls. Cycles of MPTP intoxication were repeated if the desired level of parkinsonism was not achieved in the first 7 days. Average intoxication time was 2.5 months and cumulative doses of MPTP received by all primates ranged from 13–35 mg (mean ± s.e.m.: 23.97±4 mg). Animals were filmed for 40 minutes during MPTP intoxication and stabilisation phases to quantify spontaneous locomotor activity. Once parkinsonism was satisfactory according to clinical scores and to a significant reduction in spontaneous locomotor activity (mean % ± s.e.m.: 81±6.1) that lasted at least one month, 6 primates received oral doses of L-DOPA (Modopar 125, Levodopa:Benserazide 4∶1, Roche) twice a day in order to induce dyskinesias (Fig. 1A). Primates were filmed for 6 hours on or off medication and videos were analyzed both with Ethovision® for locomotor activity quantification and The Observer® for the determination of the incidence and type of dyskinesias displayed by each subject. All LID animals received L-DOPA and a sub-cutaneous injection of amantadine, the benchmark antidyskinetic molecule, in order to validate the model used. As shown in figures 1B and 1C, drugs were tested under two different administration regimes. In acute treatments, all LID primates received a sub-cutaneous injection of a given dose of IRC or amantadine together with L-DOPA and were filmed for 6 hours (Fig. 1B). After at least 10 days washout, the same LID animals were administered L-DOPA and injected with vehicle PEG400 as a control for the acute treatments (Fig. 1B). In sub-chronic treatments, all LID primates were administered both L-DOPA and IRC-082451 or amantadine daily for 5 days and filmed on days 0 and 5 (Fig. 1C). As for acute treatments, a 10 day washout period were applied before challenging the same LID primates with the vehicle sub-chronic 5-day injections.

### LID Recordings

Quantification of L-DOPA-induced dyskinesias was performed with The Observer XT specialized software (Noldus, The Netherlands) [24]. The 6 hour recordings of animals on and off L-DOPA treatment were used to manually score the total number of dyskinesias. The analysis yielded a time-encoded event log of abnormal behaviours that allowed us to validate the pharmacokinetics of L-DOPA and the incidence of abnormal involuntary movements in all our LID primates over 6 hours. Following relevant clinical scales used in PD patients and primates [25], [26], dyskinesias were manually scored in 7 different body parts: upper limb (right or left); lower limb (right or left); trunk; neck and face. After 5 months of L-DOPA administration the LID count was stable, and animals (n = 6) were kept on a maintenance regime consisting of a daily oral dose of 600 mg of L-DOPA (mean ± s.e.m.: 566.67±55.77), and acute or sub-chronic treatments with amantadine, IRC or vehicle were alternatively administered. This same regime of L-DOPA administration was kept during the wash-out periods in between drugs.

### Euthanasia and Tissue Preparation

At euthanasia, LID primates were divided into 2 groups so that 3 were under a sub-chronic treatment with IRC-082451 and 3 under a 5-day treatment with vehicle. Because all dyskinetic animals had received IRC, amantadine and vehicle treatments over time, any permanent biochemical changes that could have been induced by amantadine were equal in all animals at the time of euthanasia and should not explain the differences observed between IRC-treated and PEG400-treated primates.

Animals were anesthetized with 10∶1 mg/kg ketamine:xylazine and blood samples were collected on the 5^th^ day of treatment with IRC-082451 or vehicle and L-DOPA, 2 hours after their administration. A lethal dose of pentobarbital was delivered before transcardial perfusion with cold 0.9% NaCl. Brains were extracted and sliced into 4 mm-thick slices using a coronal cynomolgus brain matrix (Ted Pella inc., Redding, CA, USA). Biopsy punches were taken from the anterior (AC+3) and posterior (AC-2) putamen. Punches were immersed in liquid nitrogen and kept at −80°C until prepared to be used in either RT-qPCR or HPLC-MS/MS analysis. The rest of the brain was immediately placed in ice-cold 4% paraformaldehyde for 5 days and sucrose-containing phosphate buffer gradients for cryoprotection before immunohistochemistry.

### Immunohistochemistry

Brains were sliced into 40 µm-thick sections using a freezing microtome. Sections were first incubated in 1% H_2_O_2_ for 20 minutes and for 30 minutes in phosphate buffer saline (PBS) containing 4.5% normal goat serum and 0.2% Triton-X100. Sections were then incubated for 48 h at room temperature in PBS containing 0.2% triton-X100, 3% normal serum and the appropriate dilution of the primary antibody, anti-Tyrosine Hydroxylase (TH) 1∶500 (Immunostar, Hudson, USA). After incubation in the primary anti-serum, sections were processed with the avidin-biotin peroxidase method. Unbiased stereology using the optical fractionator method was used to estimate the total number of TH positive neurons in the substantia nigra using Mercator software (Explora Nova, La Rochelle, France) as previously described [27].

### Real-time qRT-Polymerase Chain Reaction (PCR)

Putaminal brain samples were processed independently for each animal (n = 3/group). Total RNAs were extracted using guanidiumthiocyanate/phenol derived method (Trizol, Invitrogen, France) followed by digestion with RQ1 DNase (Promega, France). cDNA synthesis was performed with 2 µg of total RNA using random hexamers and a reverse transcriptase (RT2 first strand kit ref. C-03, SABiosciences). PCR was carried out with 1% of the RT product in a 20 µl reaction volume containing Platinum SYBR Green qPCR SuperMix-UDG (Invitrogen, France), 10 µM of forward and reverse primers, and 0.5% random-primed cDNA generated from 200 ng of total RNA. A MastercyclerRealplex (Eppendorf, France) was programmed for an initial denaturation step (95°C, 2 min) followed by 40 amplification cycles (95°C for 15 s, 60°C for 1 min). Expression differences were calculated using the amplification rate of the target as evaluated from serial dilutions of whole-brain cDNA and corrected for differences in cDNA input in reference to the expression level of glyceraldehyde-3-phosphate dehydrogenase (GAPDH) mRNA, assumed to be constant throughout the brain. Differences between control and experimental samples were calculated using the ΔΔCt method. All experiments were carried out on two replicates of each sample. Primer sequences were designed using Oligo 6.3 software and are reported on table S1.

### HPLC Analysis of IRC-082451 and L-DOPA Levels

Plasma samples and biopsies from cortical brain tissue were collected from three LID animals at euthanasia after 5 days of sub-cutaneous treatment with 5 mg/kg IRC-082451 and L-DOPA. Plasma samples, (50 µl) with added Terfenadin as the internal standard, were mixed at 4°C with acetonitrile containing 0.1% formic acid to precipitate the proteins. After centrifugation (2000 g for 10 min at 4°C), the supernatants were analyzed by HPLC-MS/MS for the determination of IRC-082451 levels. Samples taken from the cerebral cortex were weighted and homogenized in methanol (3 mL/g of tissue) at 4°C using a Polytron® homogenizer (Kinematica) for 60 seconds. The tissue extracts were centrifuged at 25000 g for 15 min at 4°C. The supernatants (50 µL) with Terfenadin added as the internal standard were analyzed by HPLC-MS/MS using a surveyor HPLC (Surveyor Autosampler and MS Pump, Thermo Electron®) coupled to a TSQ Quantum Ultra mass spectrometer (ThermoElectron®) equipped with an atmospheric pressure electrospray ionization source. For L-DOPA quantification, samples were prepared by precipitation of proteins in HClO4 23% perchloric acid in water (20 min at 4°C) followed by centrifugation at 2000 g for 10 min at 4°C, another cycle of centrifugation at 10000 g for 10 min at 22°C and filtration. The supernatant was kept and processed for high-performance liquid chromatography (HPLC) analysis. L-DOPA concentrations in the supernatant were determined by HPLC with electrochemical detection. The system consisted of a pump (Shimadzu model LC-10 AD, Shimadzu Europe, Munich, Germany), a refrigerated automatic injector (Famos model, Dionex, France), a reverse-phase Hypersil RP 18 analytical column (Aquasil 150×1 mm, particle size 3 µm; ThermoHypersil, les Ulis, France) and an electrochemical detector (Decade, Antec, The Netherlands) equipped with an analytical cell (type VT-03, Antec). Chromatograms were collected and treated with integration software (CLAS VP, Shimadzu, France). The mobile phase consisted of sodium dihydrogen phosphate buffer (NaH2PO4, 50 mM; Merck), octane sulfonic-1 acid sodium salt (1.7 mM; Merck), disodium ethylenediamine tetra-acetic acid (Na2-EDTA, 200 µM; Merck). The pH was adjusted to 3 with concentrated phosphoric acid (H3PO4), and 5% acetonitrile (ACN, Sigma) was added to the final solution. All solvents were filtered through Millipore filters with 0.22 µm pores (Millipore, France) before use. The mobile phase was delivered by a pump with a flow rate of 60 µl/min. The working electrode potential was +750 mV, which represented the best compromise regarding the optimum oxidation potentials of L-DOPA. The running time for each sample determination was 15 minutes.

### Imaging

#### I. Positron emission tomography scans

PET scan acquisition was performed under propofol anaesthesia (1 mg/kg/hour). Animals were placed in a PET dedicated stereotactic-like animal holder with the head resting on a mouth bar, fixed by blunt ear bars. Temperature was maintained at 37°C using a feed-back coupled heating blanket, and the respiration rate, pO_2_, pCO_2_ and cardiac rhythm were continuously monitored. ^18^F-DOPA radiotracer (6-[18F] Fluoro-L-Dopa, 200+/−30 MBq) was injected intravenously at the start of data acquisition. Imaging was performed on a non-human primate dedicated PET scanner with a 1.5 mm axial resolution 4% sensitivity, the FOCUS 220 (Siemens), using a time coincidence window of 6 ns and energy discrimination levels between 350 and 650 keV. Data were acquired during 90 min and list mode data were sorted in 9 frames of 10 min. The attenuation correction factors were measured with an external ^68^Ge source before radiotracer injection. The emission sinograms (i.e. each frame) were normalized, corrected for attenuation and radioactivity decay, and reconstructed using Fourier rebinning (FORE) and ordered subset expectation maximization (OSEM) 2D (16 subsets and 4 iterations). Data analyses were performed using the in-house built and freely available software Brainvisa (http://brainvisa.info/index_f.html). Left and right putamen were individually segmented on summed ^18^F-DOPA PET images using intensity scaling, including pixels with an intensity of 80% of the maximum intensity value within a defined volume. A spherical region (300 mm^3^) was placed in the occipital cortex as a reference region. Time activity curves representing the mean distribution of the tracer concentration (Bq/cc) within the caudate, putamen and occipital cortex in function of time were then analysed using the Patlak graphical method [28]. Equilibrium was considered to occur 30 min after tracer injection and Ki was obtained as the slope of the Patlak curve [29].

#### II. Magnetic resonance imaging

Magnetic resonance imaging (MRI) was performed on all animals shortly before or after the PET scan in order to allow precise determination of regions of interest for PET analysis. Animals were anesthetized with 10∶1 mg/kg ketamine:xylazine and placed in the magnet in a sphinx position, fixed by mouth and ear bars to a stereotactic MRI-compatible frame (M2E, France). Once in the magnet, animals were heated by a hot air flux and their temperature and respiration parameters monitored remotely.

MRI was performed on a 7 Tesla horizontal system (Varian-Agilent Technologies, USA) equipped with a gradient coil reaching 100 mT/m (300 µs rise time) and a circular radiofrequency 1H coil (12 cm inner diameter). T2-weighted images were acquired using a fast spin-echo sequence with the following parameters: TR = 4750 ms, effective TE = 62 ms, acquisition time = 16 min, FOV = 115×115 mm and matrix = 256×256 resulting in a 450×450 µm in plane resolution, 40 coronal slices, slice thickness = 1 mm.

### Statistical Analysis

#### I. Behavioural data

Analysis of variance on raw data indicated heterogeneous LID baseline from a monkey to another one (ANOVA: p = 0.0002). Since all LID animals were filmed under IRC and, after a washout period, under vehicle, each animal was compared with itself. Accordingly, the LID raw data was normalised by deriving the percentage of effect on the LID. No LID being considered as 100% of effect and the LID[PEG400] for an animal at a treatment period being considered as its 0% of effect at this period. The general formula is: LID[%effect] = 100*(LID[PEG400]-LID[raw data])/LID[PEG400]. Student’s *t* test was used to compare the percentage of effect on LIDs and TDT in relation to 0% (no effect).

#### II. RT-PCR, histology and PET

All data was analyzed with Student’s *t* test or one-way ANOVA followed by Fisher’s PLSD *post hoc* test. Data are expressed as mean ± standard error of the mean (s.e.m.).

## Results

### Effect of IRC-082451 in the Brain

#### I. Brain penetration of IRC-082451

First, plasma and brain concentrations of IRC-082451 were determined in male cynomolgus monkeys (*Macaca fascicularis*) following sub-cutaneous 5-day sub-chronic administration at 5 mg/kg/day. HPLC-MS/MS analysis of samples collected 2 h after the last injection of IRC-082451 showed a higher concentration of IRC in the brain (Cortex: 20.87±6.92 µM) than in plasma (1.16±0.33 µM), with a ratio of 18.2±2.5. This demonstrates that IRC-082451 efficiently crosses the blood-brain-barrier in non-human primates.

#### II. Potential interaction between IRC-082451 and L-DOPA metabolism

In order to rule out the possibility that the antidyskinetic effect of IRC-082451 was due to a direct interference of this molecule with the metabolism and entry of L-DOPA in the brain, the effect of injecting the IRC compound in the periphery and its bioavailability in the brain were explored *in vivo*. Blood samples were taken from all dyskinetic animals 2 hours after administration of L-DOPA and of 5 mg/kg of IRC-082451 or its vehicle. Analysis of plasma samples using HPLC separation coupled to electrochemical detection showed that there was no statistical difference between the two groups (L-DOPA concentrations = mean ± s.e.m.: LID-PEG = 276.3±8.93 ng/100 µl; LID-IRC = 303.9±48.76 ng/100 µl; N.S., p = 0.63), suggesting IRC-08451 did not markedly change L-DOPA metabolism in the periphery in parkinsonian dyskinetic primates.

**Figure 2:**
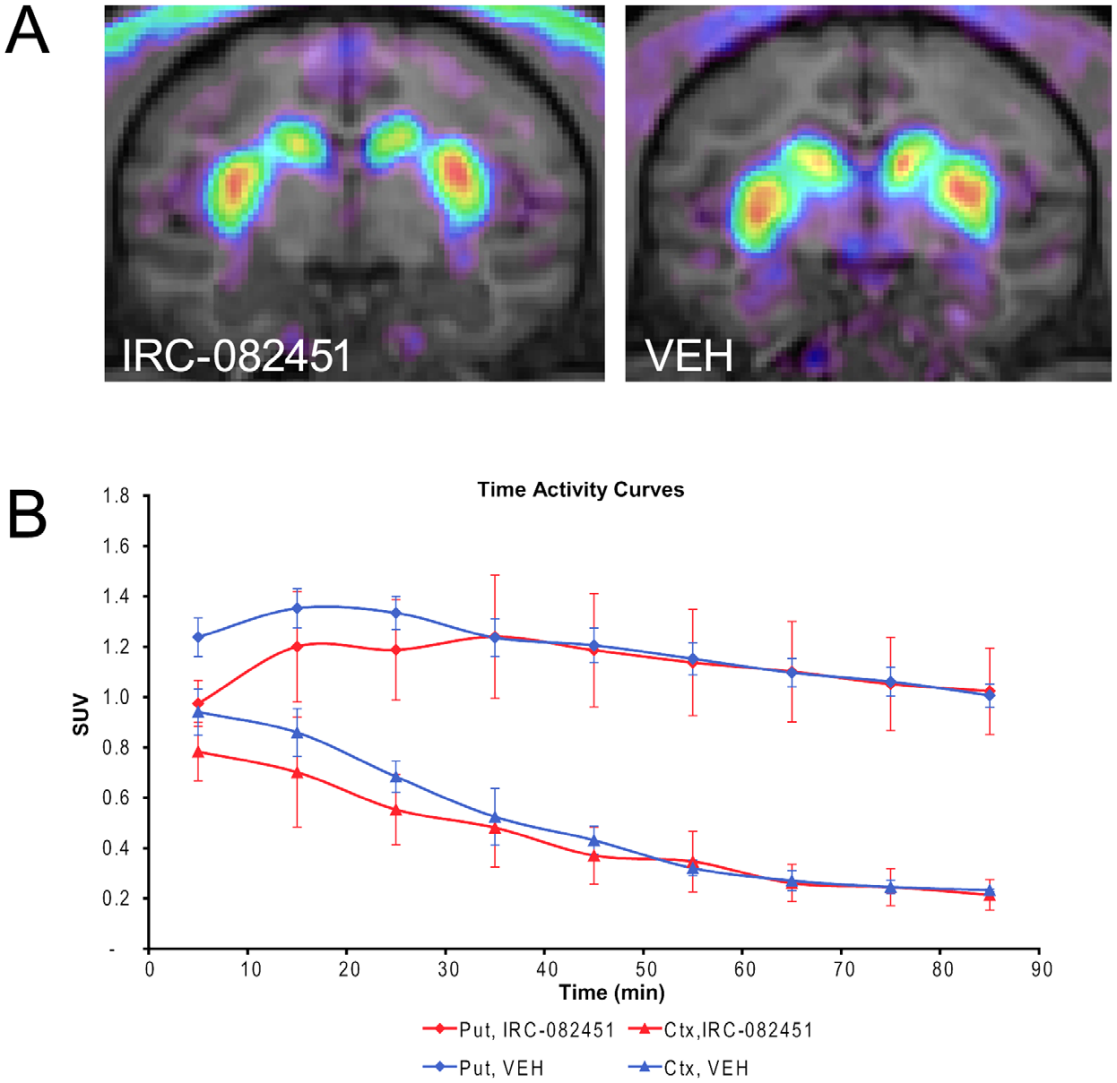
^18^F-DOPA PET studies in IRC-082451-treated and vehicle-injected healthy animals. Representative results of one subject treated with both molecules on different days taking the occipital cortex as a reference region. Coronal PET image (upper left panel) and corresponding anatomical T2-weighted MRI images (upper right panel) showing the normal accumulation of the radiotracer in the caudate and putamen (A).Time activity curves (lower panel) demonstrate there is no significant difference between treatments in either the caudate-putamen complex (Put) or the occipital cortex (Ctx) curves. Data are expressed as mean ± s.e.m.

In order to further study such potential interference in the brain, a functional assessment by PET was performed in parallel in a separate group of primates. Three healthy animals received a sub-cutaneous injection of either 5 mg/kg IRC-082451 or its vehicle PEG400 2 hours before being anesthetized for the imaging session. ^18^F-DOPA radiolabelled tracer was injected intravenously at IRC C_max_ and the acquisition lasted 90 minutes. PET images were co-registered with anatomical MRI images in order to determine regions of interest in the striatum and a region of reference in the occipital cortex. Patlak analysis revealed that the influx constant (Ki) of the radioactive tracer in striatal dopaminergic terminals was not different in animals that had been injected with IRC-082451 and those injected with vehicle and that accumulation of ^18^F-DOPA over time was comparable as shown by the images reported in figure 2A. In fact, the Ki coefficients (∼0.016 min^−^1 in normal intact NHP) calculated from the regions of interest drawn in the caudate and putamen of the right and left hemispheres were not significantly different amongst animals regardless of the treatment received (Ki ± s.e.m.: PEG400 = 0.015±0.001; IRC-082451 = 0.017±0.001) (Fig. 2B). This indicates IRC-082451 acts on dyskinesias through a mechanism different from the direct interference with L-DOPA brain uptake and/or metabolism.

### Effect of Amantadine on Dyskinesias and Locomotor Behaviour

**Figure 3:**
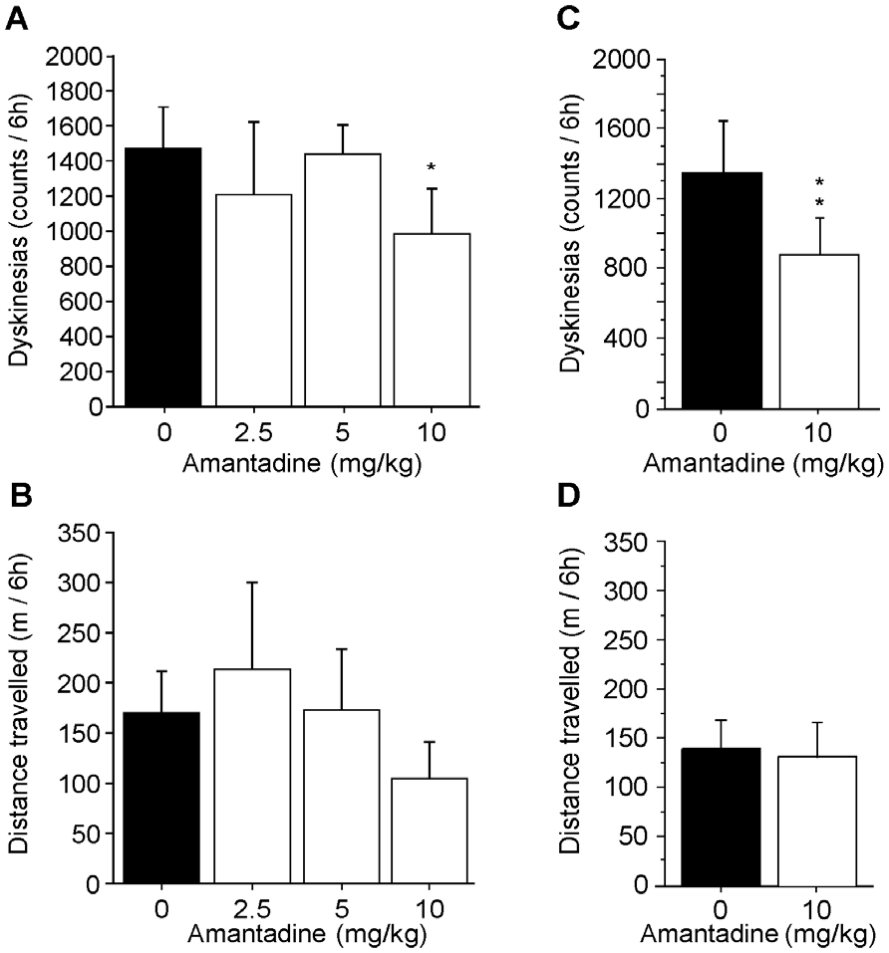
Effect of amantadine treatment on motor behaviour. Three different doses of amantadine were acutely injected together with L-DOPA and a significant antidyskinetic effect was observed at the 10 mg/kg dose (A). Ethovision analysis of the total distance travelled (TDT) shows there is no effect on locomotor activity of either dose of amantadine tested (B). Sub-chronic 5-day treatment with 10 mg/kg amantadine further confirms the antidyskinetic efficacy of the compound (C) and the lack of significant effect on locomotor activity compared to vehicle treatment (D). Data are presented as mean ± s.e.m. Unpaired Student *t* test (*, p<0.05).

Although the primate model chosen for this study has been extensively used to evaluate therapeutic strategies for PD, we wanted to confirm that our dyskinetic primates responded to treatment with amantadine, the NMDA glutamate receptor antagonist most widely used in the clinic to reduce LIDs [18]. Three doses of amantadine were tested acutely based on published results in non-human primates [30], [31], [32], [33]. Locomotor activity was quantified using Ethovision software and the incidence of abnormal movements was rated using The Observer software. All LID animals were given L-DOPA and an acute injection of either vehicle or a given dose of amantadine (2.5, 5 and 10 mg/kg) respecting a 10-day washout period between treatments. Results indicated that amantadine significantly reduced dyskinesia score by approximately 30% only at the 10 mg/kg dose (p<0.05) (Fig. 3A). No significant effect was observed on locomotor activity for all amantadine doses as compared to vehicle (Fig. 3B). Based on these results, the 10 mg/kg dose was tested on a sub-chronic 5-day regime. Results also showed a significant effect of 39% (p<0.001) on dyskinesias by day 5 (Fig. 3C) and no effect on locomotor activity (Fig. 3D). Overall, this suggests that LID primates were responsive to a validated antidyskinetic agent and were suitable for investigating the effect of the novel compound IRC-082451.

### Effect of IRC Treatment on Dyskinesias and Locomotor Behaviour

**Figure 4:**
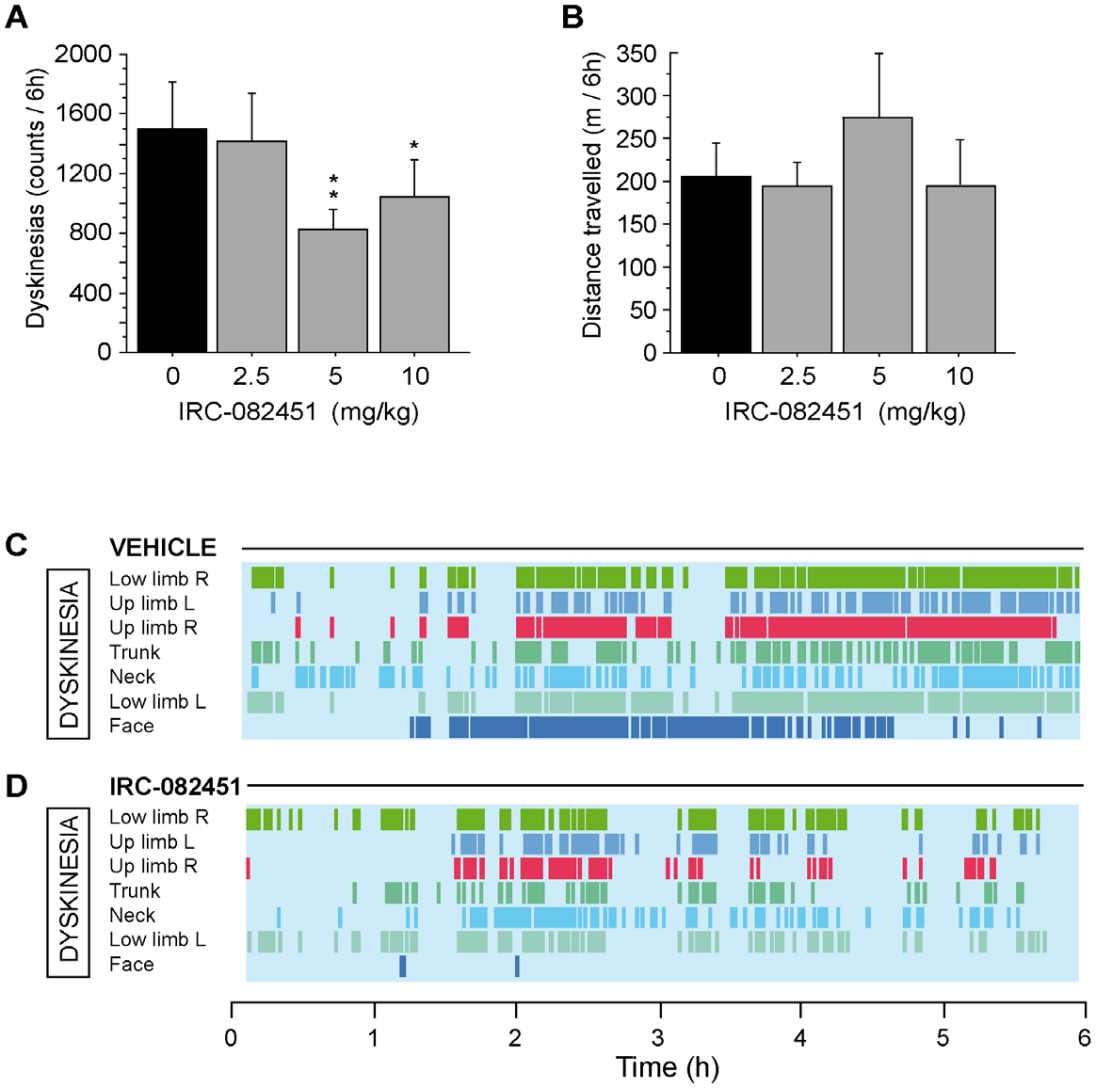
Time-encoded video-based analysis of motor behaviour after acute treatment with escalating doses of IRC-082451. Quantification of L-DOPA-induced dyskinesias at 2.5, 5 and 10 mg/kg IRC-082451 compared to vehicle (0)(A). Total distance travelled (TDT) in meters as measured by Ethovision at different IRC doses compared to vehicle (B). Visualization of dyskinesia count output from The Observer software under vehicle (C) and after an acute 5 mg/kg IRC treatment (D). Horizontal lines represent a part of the body displaying dyskinesias whereas vertical coloured lines represent an individual dyskinetic event over time. Data are expressed as mean ± s.e.m. Unpaired Student *t* test (*, p<0.05; **, p<0.01).

Figure 4A summarizes the effect observed on dyskinesias for all acute doses of IRC-082451 tested in our LID primates. The 2.5 mg/kg dose had no beneficial effect on the number or type of dyskinesias following L-DOPA treatment. In contrast, a significant 43% reduction of the total number of dyskinesias (p<0.01) was observed at the 5 mg/kg dose suggesting an antidyskinetic effect of the compound. Animals also responded acutely to the 10 mg/kg dose showing a 25% reduction in dyskinesias (p<0.05) (Fig. 4A). Figure 4B shows there was no significant effect of IRC-082451 on spontaneous locomotor activity across all acute treatments tested. An example of the time-encoded analysis of dyskinesias in a single animal using The Observer software is reported in figures 4C (PEG) and D (IRC). Statistical analysis showed there was no significant correlation between the antidyskinetic effect of IRC-082451 and a specific body part scored or a specific time after administration over the 6 hours of film (data not shown).

**Figure 5:**
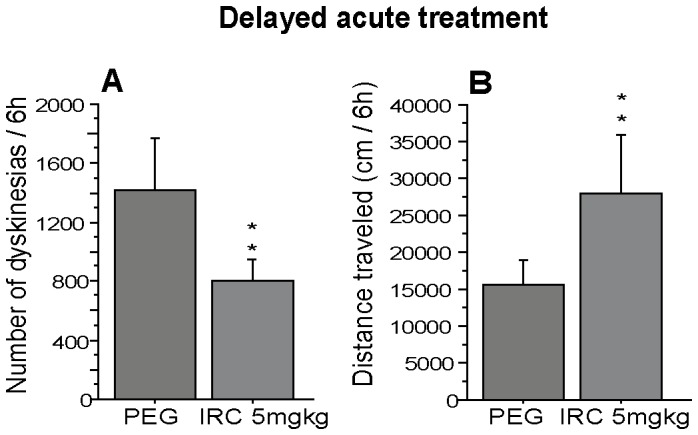
Effect of a delayed acute treatment with IRC-082451 on locomotor activity. A single dose of 5 mg/kg of IRC was given 2 hours before administration of L-DOPA and the total number of dyskinesias and distance travelled on the same day are presented. A significant reduction in LID count was observed (A) together with an increase in spontaneous distance travelled (B) compared to the same animals under vehicle treatment. Data are presented as mean ± s.e.m. Unpaired Student *t* test (**, p<0.01).

**Figure 6:**
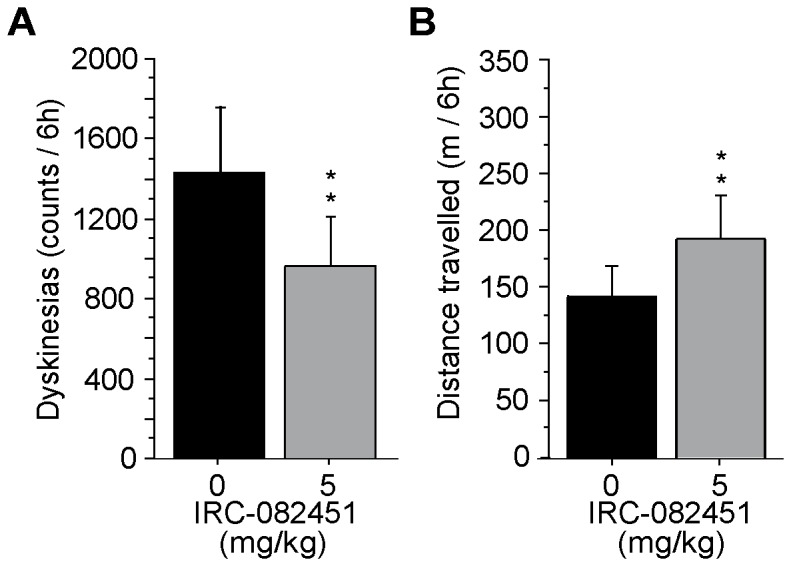
Effect of a sub-chronic treatment with IRC-082451 on locomotor activity. The 5-day sub-chronic treatment with 5 mg/kg IRC was given concomitantly with L-DOPA and the total number of dyskinesias and distance travelled on day 5 are presented. A significant reduction in LID count was observed (A) together with an increase in spontaneous TDT (B) compared to the same animals under vehicle treatment. Data are presented as mean ± s.e.m. Unpaired Student *t* test (**, p<0.01).

Although no significant difference was found between the antidyskinetic effect of IRC-082451 at 5 and 10 mg/kg when injected acutely (p<0.5), the 5 mg/kg dose showed a tendency to further reduce the incidence of dyskinesias. Interestingly, The Observer analysis highlighted that the incidence of dyskinesias begun to lower 2 hours after the administration of 5 mg/kg of IRC-082451. In order to test whether this “delayed” effect of IRC was due to its reaching C_max_ at a slower rate than L-DOPA, a different acute treatment was implemented for this dose. All dyskinetic primates received 5 mg/kg of IRC or VEH at time 0 and L-DOPA 2 hours later. Animals were then filmed for 6 hours. Results reported in figure 5A show that a significant 43% reduction in dyskinesias (p<0.01) was then achieved acutely together with a significant 42% increase in locomotor activity (p<0.04; fig. 5B). The fact that IRC and L-DOPA did not reach peak effect at the same time could be due to the pharmacokinetics of each compound and to the different administration routes used for IRC (s.c.) and L-DOPA (p.o.). Based on these results, the 5 mg/kg dose was chosen to perform a 5-day sub-chronic treatment in all LID animals. As shown in figure 6A, sub-chronic treatment with the 5 mg/kg dose of IRC-082451 had a significant antidyskinetic effect (p<0.001) and, more importantly, locomotor activity was significantly increased by 35% (p<0.01) on day 5 of treatment on all LID primates (Fig. 6B).

**Figure 7:**
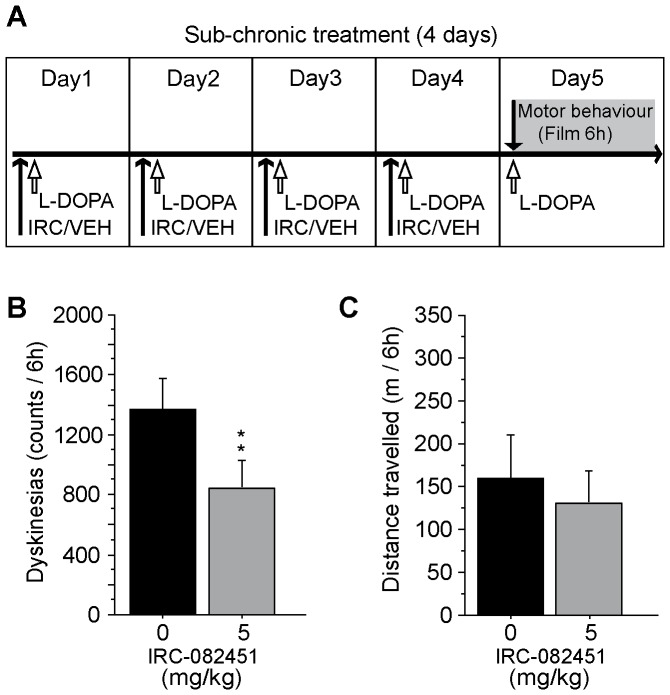
Effect of a 4-day treatment with IRC-082451 (5 mg/kg/day). The sub-chronic treatment was given concomitantly with L-DOPA for 4 days and animals were filmed on the 5^th^ day (A). Total number of dyskinesias (B) and total distance travelled (C) over 6 h of film are presented and a significant reduction in LID counts is observed. Data are expressed as mean ± s.e.m. Unpaired Student *t* test (**, p<0.01).

We were then curious to know whether the IRC molecule conferred protection 24 hours after the last administered dose. After a 10-day washout, we proceeded to a 4 day treatment with L-DOPA and 5 mg/kg IRC-082451 and filmed all LID animals on the 1^st^ and 5^th^ day (Fig. 7A). Interestingly, the treatment continued to be effective on dyskinesias (p<0.005) (Fig. 7B) although no increase in locomotor activity was observed (Fig. 7C).

Finally, the administration of IRC-082451 or its vehicle PEG400 in the absence of L-DOPA had no effect on spontaneous locomotor activity (data not shown).

### Post-mortem Characterization of the Effect of IRC-082451 Treatment on Dyskinesia

#### I. IRC-082451 effects on gene expression

Quantitative RT-PCR assays were performed for several different genes previously identified in the literature as key players in pathways involved in dyskinesias in animal models of PD. Primers were designed for the transcriptional regulators FosB and cFOS, the glutamate ionotropic receptor (GRIN2B/NR2B), the homer homologue 1 (Homer), the proenkephalin (PENK1), the preprodynorphin (PDYN), the glutamic acid decarboxylase 1 (GAD67), the serotonin receptor type 1A (5HTR1A), the metabotropic glutamate receptor type 5 (mGluR5) and the activity-regulated cytoskeleton-associated protein (ARC) (see Table S1).

**Figure 8:**
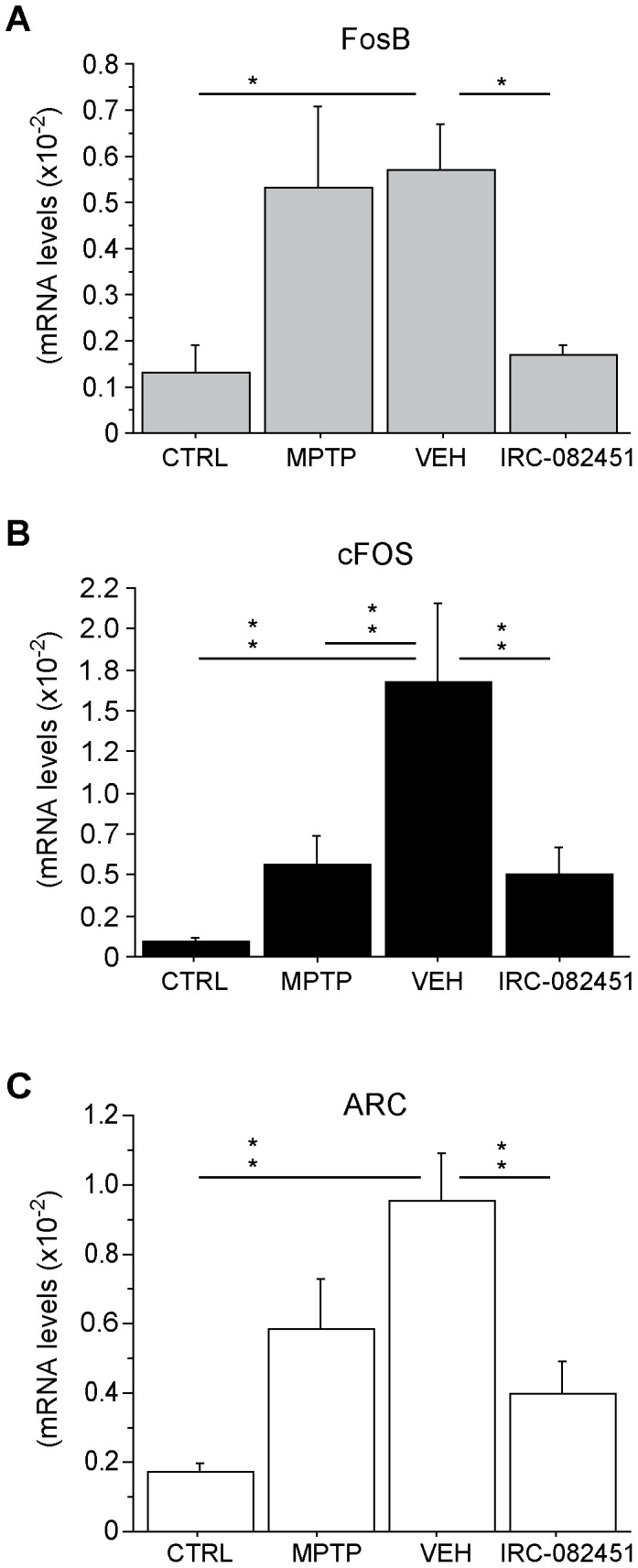
RT-qPCR analysis of putaminal brain samples in healthy controls (CTRL), parkinsonian untreated (MPTP), dyskinetic vehicle-treated (VEH) and dyskinetic IRC-082451-treated non-human primates. Significant differences in mRNA levels were detected for FosB (A), cFOS (B) and ARC (C) when comparing dyskinetic IRC-treated and dyskinetic vehicle-treated animals. Data are expressed as mean ± s.e.m. One way ANOVA followed by a *post hoc* Fisher’s PLSD test (*, p<0.05; **, p<0.01).

ANOVA indicated no statistically significant differences between groups for seven out of the ten genes tested (see figure S1). This is most likely due to the limited number of animals included in each group (n = 3) and the limited amount of brain tissue that can be recovered for PCR experiments while preserving some tissue for histological analysis. However, in agreement with most of the literature dealing with dyskinesias, significant changes were observed in FosB and cFOS mRNA levels in the putamen of parkinsonian dyskinetic animals that were corrected after sub-chronic treatment with IRC-082451. In fact, as expected from the animal model, FosB mRNA expression levels were higher in the putamen of LID animals under chronic L-DOPA and treated with vehicle when compared to healthy controls (p<0.03). IRC treatment fully reversed such increase (p<0.02 vs VEH) (Fig. 8A). cFOS levels were similarly higher in vehicle-treated dyskinetic animals than in MPTP parkinsonian animals and healthy controls (p<0.001), an effect fully reversed by IRC-082451 sub-chronic treatment (p<0.01 vs VEH) (Fig. 8B).

Interestingly, changes in a novel gene were also found in this model. ARC mRNA levels were significantly increased in the putamen of dyskinetic vehicle-injected primates compared to healthy controls (p<0.001) while a significant reduction of ARC was observed in dyskinetic IRC-082451-treated animals (p<0.005 vs VEH) (Fig. 8C). A MANOVA analysis on the expression levels of all the above mentioned genes and the responsiveness to the IRC-082451 treatment in dyskinetic animals was carried out. Dyskinesia counts of the same animals on L-DOPA and either vehicle or IRC-082451 were used to calculate a percentage of response to the antidyskinetic treatment. Supporting the RT-qPCR data, ARC significantly correlated with responsiveness to the IRC-082451 treatment (p<0.05).

#### II. Characterization of the dopaminergic lesion

**Figure 9:**
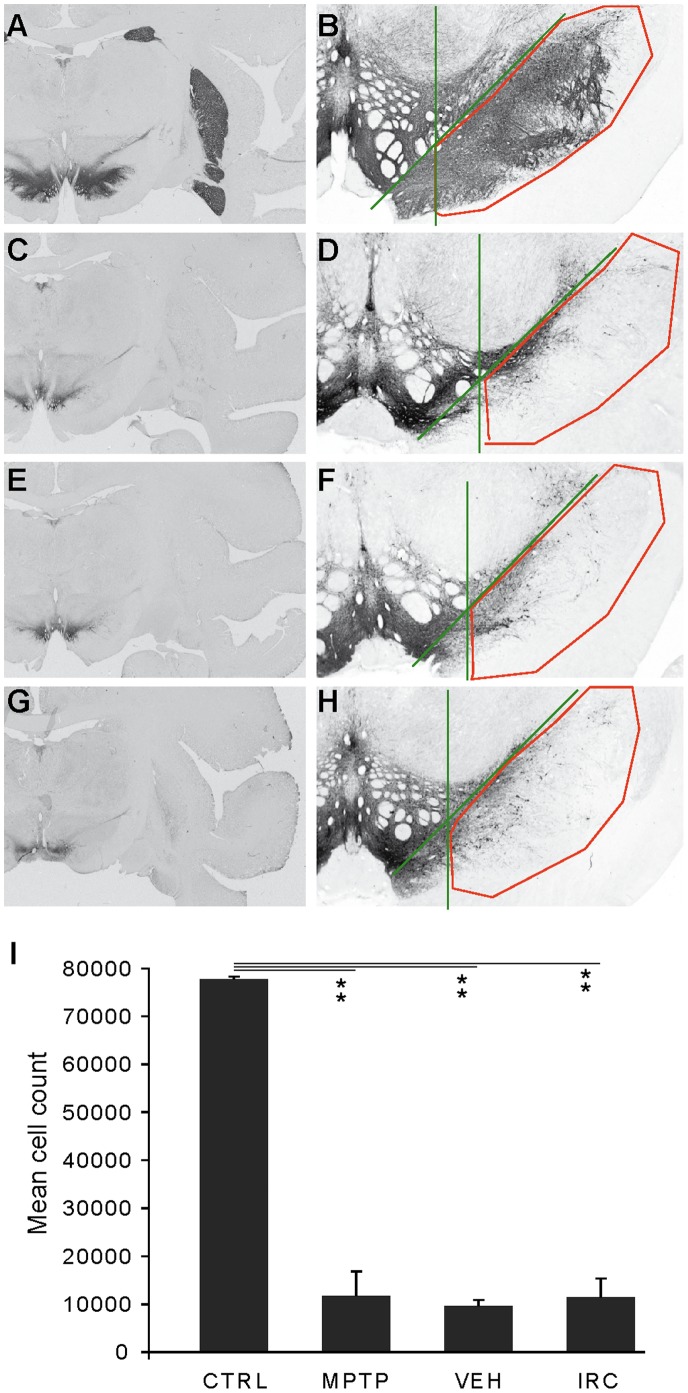
Histological characterization of the nigrostriatal dopaminergic pathway in the all primate brains. Left column: representative coronal sections of healthy (CTRL, A), parkinsonian (MPTP, C), dyskinetic vehicle-treated (VEH, E) and dyskinetic IRC-treated (IRC, G) primate brains stained for tyrosine hydroxylase. Right column: areas of the substantia nigra where stereological counts were performed drawn on representative samples for each group of animals (B, CTRL; D, MPTP; F, VEH; H, IRC). Lower panel: histogram displaying TH-positive neuron number in the substantia nigra (I). Data are expressed as mean cell counts ± s.e.m. Unpaired Student *t* test (**, p<0.01).

Figure 9 shows representative brain sections of monkeys in each treatment group stained for TH in order to assess the level of dopaminergic deafferentation in the striatum (Fig. 9A, C, E, G) and cell loss in the substantia nigra pars compacta (Fig. 9B, D, F, H). Healthy controls presented a strong dark signal in the striatum and SNc that was severely reduced (∼90%) in animals that underwent MPTP intoxication, suggesting a similar level of dopamine depletion across groups. Stereological counts in the SN confirmed there were no significant differences in the number of TH-positive neurons between MPTP parkinsonian controls, LID-vehicle-treated and LID-IRC-treated animals (Fig. 9I), whereas all of these groups were significantly (p<0.001) different from healthy controls.

## Discussion

The present study demonstrates the efficacy of the novel multitargeting hybrid molecule, IRC-082451, in reducing the incidence of L-DOPA-induced dyskinesias in the MPTP primate model of PD. The antidyskinetic effect of this compound was significant acutely, after a sub-chronic 5-day treatment period and even 24 hours after the last administered dose. The effect of IRC was compared to that of amantadine at three incremental doses. Both compounds were clearly ineffective at the 2.5 mg/kg dose, IRC and not amantadine was efficacious at the 5 mg/kg dose and both compounds showed an antidyskinetic effect at the 10 mg/kg dose. The antidyskinetic effects of 5 and 10 mg/kg doses of IRC-082351 were not significantly different suggesting that the maximal beneficial effect of this compound was reached at 5 mg/kg in our LID model. The behavioural analysis carried out consisted of a manual continuous 6 h neurological scoring of LIDs following L-DOPA ingestion and a concomitant automated quantification of the spontaneous locomotor activity displayed by primates during the same 6 hours. This made it possible to observe that the antidyskinetic effect of IRC-082451 was associated with a significant increase of the beneficial effect of L-DOPA on the total distance travelled after a 5-day treatment with IRC-082451. Detailed analysis of leg, arm, trunk, neck and oro-lingual dyskinesia suggested there was no exclusive effect of IRC-082451 on dyskinesia affecting a particular body part nor a particular type of dyskinesia. These results are consistent with and further support those obtained in dyskinetic 6-OHDA rats where the antidyskinetic properties of acute and sub-chronic treatments of IRC-082451 have been recently reported [17]. From a clinical standpoint, our behavioural analysis suggests that IRC-082451 could at least partially alleviate LIDs in PD patients under chronic L-DOPA therapy and may also allow the reduction of L-DOPA doses without compromising its beneficial effect on akinesia.

Several compounds have been tested for their potential effects against LIDs in the MPTP model of PD in non-human primates. They cover a variety of therapeutic targets such as glutamatergic receptors (amantadine) [30], [32], mu-opioid receptors (ADL5510) [34], nicotinic receptors [35] and the serotonin transporter blocker (3,4-methylenedioxymethamphetamine) [36]. The heterogeneity in the choice of animal model, the administration route of the compounds and the functional assessment used in these studies makes it difficult to reliably compare the efficacy of IRC-082451 to that of such potential antidyskinetic agents. However, treatment with the reference molecule amantadine confirmed that our LID primates were responsive to an antidyskinetic agent as expected. Interestingly, the therapeutic effect of IRC-082451 against LIDs was accompanied by an increased beneficial effect of L-DOPA on locomotor activity. The effect of IRC-082451 on L-DOPA-induced reduction of akinesia is not due to a direct effect on locomotion since the IRC-treatment alone did not change the locomotor activity of MPTP primates in the absence L-DOPA. Moreover, an interesting property of IRC-082451 resides in its pharmacodynamic characteristics. We showed that following a 4-day treatment, its efficacy lasted for a further 24 h, allowing significant reduction of LIDs on the 5^th^ day. To the best of our knowledge, such delayed effect has never been reported for other compounds, suggesting that in a clinical perspective, IRC-082451 treatment might provide relatively stable effects even in cases of poor patient compliance.

A reduction in the incidence of dyskinesias might simply be due to an acceleration of L-DOPA elimination or an alteration of its cerebral metabolism, leading to reduced dopamine synthesis. However, this is unlikely for IRC-082451, since the compound decreased the number of LIDs while increasing locomotion, indicating that the L-DOPA treatment remained centrally efficacious. In addition, an *in vivo* PET imaging study demonstrated that there was no significant interaction between IRC-082451 and ^18^F-DOPA suggesting that the effect on locomotor activity and dyskinesia is unlikely related to the compound’s direct interaction with L-DOPA or dopamine cerebral metabolism. These findings were further confirmed by the *in vivo* HPLC dosage that showed that L-DOPA plasma levels were similar in animals treated with IRC-082451 or its vehicle.

As a multi-targeting molecule, the IRC compound possesses several properties that cooperate to interfere with several signalling paths involved in LID. IRC has been shown to act as an anti-oxidant and a sodium channel blocker, thus protecting neurons from two plausible mechanisms underlying the pathophysiology of LIDs: excitotoxicity and free radical-induced damage resulting in dysfunctional mitochondria [16]. In fact IRC-082451 can protect mitochondria from MPP+ and Ca^2+^ induced-permeability transition and cytochrome c release, all events linked to the triggering of apoptosis. Thanks to its mitochondrial protective properties, this treatment could compensate for the chronic oxidative stress induced by L-DOPA during its metabolism and the reduced mitochondrial energy output resulting from dyskinesia [37]. Moreover, indirect regulation of glutamate release, a neurotransmitter majorly involved in dyskinesias, is also mediated by the IRC compound *in vitro* and *in vivo* thanks to its sodium channel blocker properties. Finally, unlike many proposed antidyskinetic treatments, the mechanism of action of this compound does not directly target dopaminergic, serotoninergic or glutamatergic receptors [16], thus offering a new alternative therapeutic strategy against dyskinesias.

Although the underlying mechanisms of action of IRC-082451 against LIDs are yet to be fully deciphered, post mortem analyses of the striatum of dyskinetic animals suggest that sub-chronic IRC treatment can reverse some of the molecular anomalies associated with LID. We observed a reduction in the upregulation of FosB and cFos mRNA in dyskinetic animals that were responsive to the treatment with this molecule as compared to vehicle-treated dyskinetic controls. We found that the activity-regulated cytoskeleton-associated (Arc) gene was strongly upregulated upon dopaminergic depletion and dyskinesias and that its expression levels were normalized after IRC-082451 treatment. Experiments in dyskinetic rats treated with chronic L-DOPA have shown upregulation of ARC mRNA and protein in dynorphin-containing striatonigral neurons that correlated to the severity of abnormal movements [37], [38]. In line with our findings, the effect of IRC-082451 on ARC mRNA expression in 6-OHDA rats with LIDs has also been recently published [17]. Moreover, in our study, FosB, a known marker of LIDs, was increased in dyskinetic vehicle-treated animals and downregulated in dyskinetic IRC-treated primates. A significant correlation was found between ARC and FosB mRNA levels. ARC appears as a key molecular player in the reduction of dyskinesia in both rodent and primate models of PD. The induction of ARC mRNA and synaptic localization of the protein are both regulated by NMDA receptors as shown by experiments on long-term potentiation, learning and memory in rodents (reviewed in [39], [40], [41]). Several groups have described the role of synaptic remodelling in LIDs, comparing them to an aberrant form of motor “patterning” or “learning” [42], [43], [38], alterations of the glutamatergic synapse being central in the physiopathology of LIDs [44]. ARC is associated to NMDA glutamatergic receptors as well as regulating AMPA receptor trafficking [41], [38], [45]. It forms a protein complex with PSD95 and CAMKII and has been localized to the PSD in dendritic spines (see [39]). ARC could have a dual function acting indirectly on glutamate release as well as enhancing synaptic plasticity. Further studies are necessary to elucidate the role of this protein in LIDs and to determine the precise mechanism through which the IRC molecule exerts its antidyskinetic effect in preclinical models of PD.

In summary, thanks to the unique combination of motor activity and involuntary movement recording analysis, our results show that IRC-082451 treatment enhances the benefit of L-DOPA pharmacotherapy by reducing LIDs and increasing spontaneous locomotion. Plasma dosages and PET imaging experiments rule out the possibility that the reduction in dyskinesias is due to a reduced bioavailability of L-DOPA in the primate brain. The behavioural effect IRC-082451 is related to the attenuation of the aberrant molecular changes likely related to abnormal glutamate transmission in the striatum, including increases in ARC, cFos and FosB expression. This supports the hypothesis that IRC-082451 has an effect on synaptic remodelling that in turn regulates the long-term motor maladaptive alterations caused by LIDs, thus linking changes in synaptic connectivity with behavioural outcome.

## Supporting Information

Figure S1 **RT-qPCR analysis of putaminal brain samples in healthy controls (CTRL), parkinsonian untreated (MPTP), dyskinetic vehicle-treated (VEH) and dyskinetic IRC- 082451-treated non-human primates for seven different genes implicated in dyskinesias.** F and p values are reported for each of the genes tested: the glutamate ionotropic receptor (GRIN2B/NR2B), the homer homologue 1 (Homer), the proenkephalin (PENK1), the preprodynorphin (PDYN), the glutamic acid decarboxylase 1 (GAD67), the serotonin receptor type 1A (5HTR1A), the metabotropic glutamate receptor type 5 (mGluR5) and the activity regulated cytoskeleton-associated protein (ARC). Data are expressed as mean ± s.e.m. One way ANOVA showed no statistically significant differences between groups for these markers. The F and p values of the ANOVA are indicated on the top left corner of each panel.
(TIF)

Table S1 **Sequences of all primers used for qPCR analysis.** Sequences for forward (F) and reverse (R) primers are reported for all genes explored including PPIA, GAPDH, PENK1, GAD67, ARC, PDYN, cFos, FosB, GRIN2B, Homer, HTR1A, and GRM5. Primer pairs were generated using the Oligo 6.3 software.
(TIF)
