# Correction: IRC-082451, a Novel Multitargeting Molecule, Reduces L-DOPA-Induced Dyskinesias in MPTP Parkinsonian Primates

**DOI:** 10.1371/annotation/2efe0ad6-ebf7-434b-94c3-f4225c4d31d7

**Published:** 2013-01-30

**Authors:** Romina Aron Badin, Brigitte Spinnewyn, Marie-Claude Gaillard, Caroline Jan, Carole Malgorn, Nadja Van Camp, Frédéric Dollé, Martine Guillermier, Sabrina Boulet, Anne Bertrand, Marc Savasta, Michel Auguet, Emmanuel Brouillet, Pierre-Etienne Chabrier, Philippe Hantraye

There is an error in the Competing Interests statement. The correct statement is: Co-authors Auguet M, Chabrier PE, Spinnewyn B are employed by IPSEN Innovation. The research reported in this manuscript has been funded in part by IPSEN Innovation (http://www.ipsen.com/fr). BN82451 is patented (Pätent portfolio made up of different family of patents: Novel of 2-(iminomethyl)amino-phenyl derivatives intermidiates; e.g. US6586454; 5-membered heterocycle derivatives: e.g. WO01/26656, WO2005/035510; Use of thiazole derivatives for preparing a medicament for protecting the mitochondria: e.g. WO 03/009843; Thiazoles derivatives for the treatment of dyskinesia: e.g. WO2007/006942 and Thiazoles derivatives for the treatment of restless legs syndrome: WO2007/006941) and is being developed by Ipsen. This does not alter our adherence to all the PLOS ONE policies on sharing data and materials.

