# Supplementary figures and images for: IRC-082451, a Novel Multitargeting Molecule, Reduces L-DOPA-Induced Dyskinesias in MPTP Parkinsonian Primates
Source: PLoS One. 2013 Jan 3;8(1):e52680. doi: 10.1371/journal.pone.0052680 (PMC3536787; doi:10.1371/journal.pone.0052680)

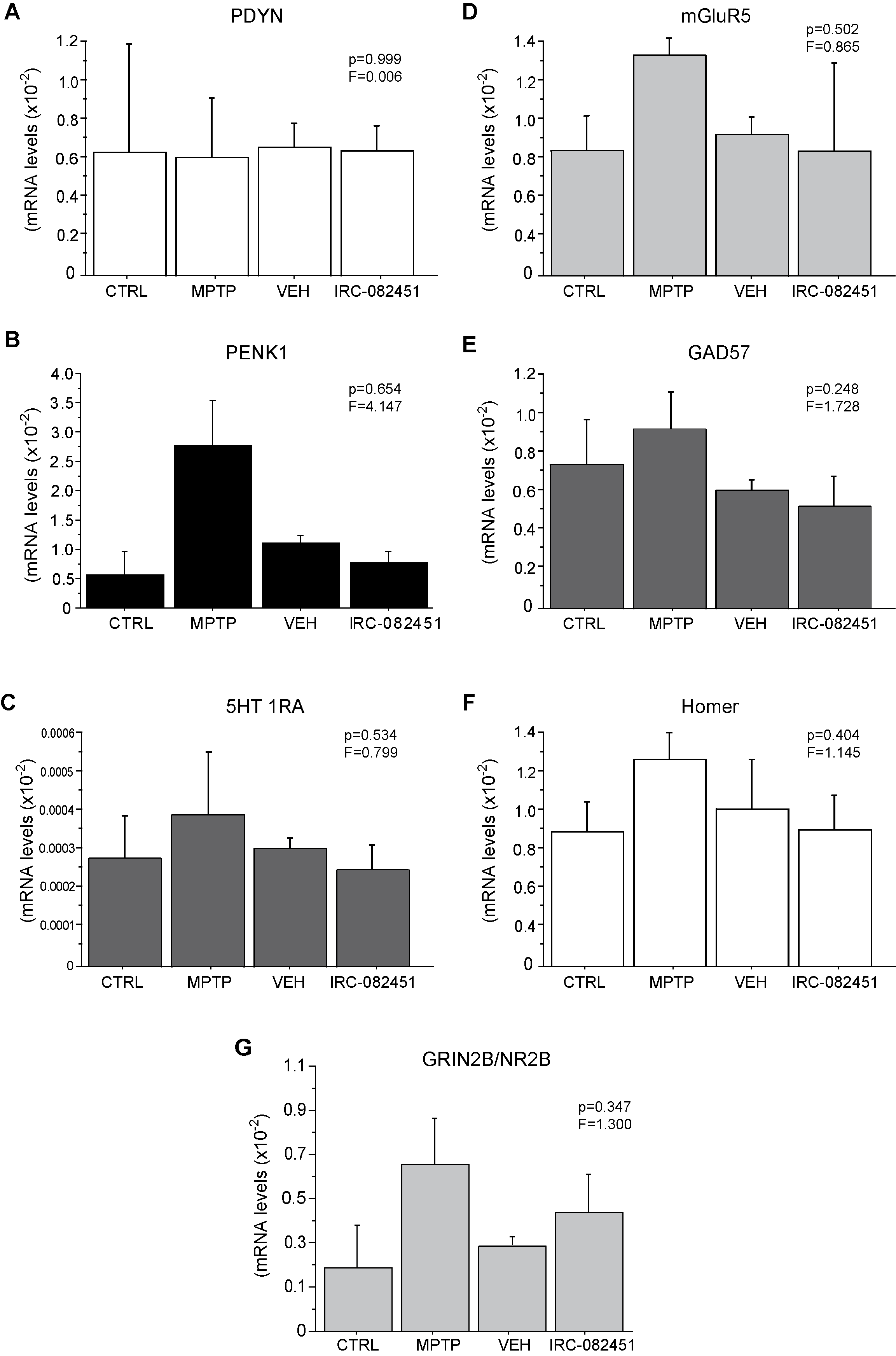

Supplement: Figure S1 — RT-qPCR analysis of putaminal brain samples in healthy controls (CTRL), parkinsonian untreated (MPTP), dyskinetic vehicle-treated (VEH) and dyskinetic IRC- 082451-treated non-human primates for seven different genes implicated in dyskinesias. F and p values are reported for each of the genes tested: the glutamate ionotropic receptor (GRIN2B/NR2B), the homer homologue 1 (Homer), the proenkephalin (PENK1), the preprodynorphin (PDYN), the glutamic acid decarboxylase 1 (GAD67), the serotonin receptor type 1A (5HTR1A), the metabotropic glutamate receptor type 5 (mGluR5) and the activity regulated cytoskeleton-associated protein (ARC). Data are expressed as mean ± s.e.m. One way ANOVA showed no statistically significant differences between groups for these markers. The F and p values of the ANOVA are indicated on the top left corner of each panel. (TIF) [file pone.0052680.s001.tif]
